# Effect of Carbon Dioxide Laser Ablation Followed by Intralesional Steroids on Keloids

**DOI:** 10.4103/0974-2077.79176

**Published:** 2011

**Authors:** Gaurav A Garg, Prajct P Sao, Uday S Khopkar

**Affiliations:** *Department of Dermatology, Seth GS Medical College and KEM Hospital, Mumbai, India*

**Keywords:** Carbon dioxide laser, intralesional steroid, keloid

## Abstract

**Abstract:**

Keloid is a difficult-to-treat condition and an ideal treatment modality is not available. Carbon dioxide (CO_2_) laser is one of the modalities to treat keloids.

**Aim:**

To evaluate the effect of CO_2_ laser ablation followed by intralesional steroids on keloids.

**Settings and Design:**

This was a prospective, single-center, uncontrolled, open study.

**Materials and Methods:**

Twenty-eight patients having 35 keloids were included in the study. Keloids were ablated or excised with CO_2_ laser followed by intralesional steroid 3-4 weeks apart for 6 months. Results were evaluated after 6 months of stopping of intralesional steroids.

**Statistical Analysis:**

Fisher’s exact test was applied for obtaining difference in recurrence rate of regular and irregular patients.

**Results:**

Thirteen patients followed up regularly for intralesional steroids. During 6 months of follow-up after stoppage of steroids, only two patients showed recurrence. Ten patients were irregular for intralesional steroids and seven of them showed recurrence. Difference in recurrence rate of regular and irregular patients was significant.

**Conclusion:**

Only CO_2_ laser ablation is not sufficient for halting the pathogenesis of keloid formation.We therefore conclude that CO_2_ laser followed by intralesional steroid is a useful therapeutic approach for the treatment of keloids; however, patients need to be observed for recurrence over the next 1 year.

## INTRODUCTION

Keloids are benign hyperplasias which may or may not be preceded by injury. They are refractory to treatment most of the times. Intralesional corticosteroids,[1] topical retinoic acid,[2] topical imiquimod cream,[3] surgery,[4] cryotherapy,[5] laser[6] and silicon sheeting[7] are mainly used for their treatment. We assessed the effect of carbon dioxide (CO_2_) laser ablation followed by intralesional steroid on 35 keloids of 28 patients.

## MATERIALS AND METHODS

Twenty-eight patients having 35 keloids were included in the study. A detailed clinical history and examination were done for each patient. Diagnosis of keloid was done on clinical basis. Size of each keloid was noted with the help of a scale. The patients with keloids of size less than 10 cm in any dimension, any duration and with or without any treatment taken in the past were included in the study. However, patients of age < 12 years, pregnant females and infected or secondary changes on surface of keloid (e.g., excoriation/ eczema) were not included in the study. Written informed consent was taken. Photographs were taken on every follow-up e.g., after 3-4 weeks.

**Figure 1:**
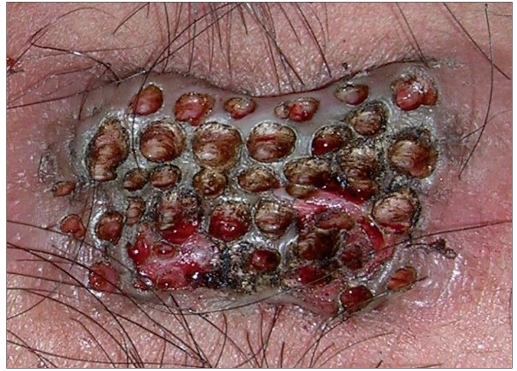
Multiple puncture technique. Small full thickness punctures were created throughout the keloid tissue with a gap of 1-2 mm between the two punctures

**Figure 2:**
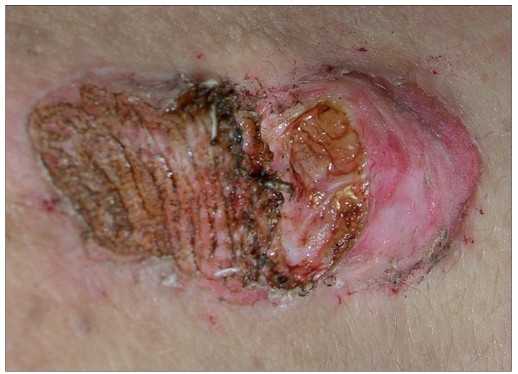
Excision from base of keloid tissue

The CO_2_ laser machine used (model no CL20, Sunny Optoelectronic Co. Ltd, Shanghai, China) has continuous, repetitive and super-pulse mode. Energy per second (power) ranges from 0.5 to 15 W. Field block anesthesia(2% lignocaine with epinephrine 1:200000 infiltrated around the margins of the keloid) was given prior to the procedure. Patients were treated on the basis of the size of lesions. Smaller lesions were vaporized using super pulse with 15 W power. Larger lesions were treated in single or stagewise manner by multiple puncture technique or by excision. In multiple puncture technique, small full thickness punctures were created throughout the keloid tissue with a gap of 1-2 mm between the two punctures by CO_2_ laser. Super pulse (for firm keloid) or continuous (for hard keloid) mode was used with 15 W power [Figure 1]. In excision, keloid tissue was excised from base with super pulse (for firm keloid) or continuous (for hard keloid) with a power output of 15 W [Figure 2]. Keloid was ablated till upper reticular dermis, indicated by yellow to light brown (faun) color change.[8] Orally, 500 mg of tab amoxycillin with 125 mg clavulanic acid was given two times a day for 7-14 days depending on the healing of the lesion. Topically mupirocin cream was given till the healing of the lesion. Patients were advised to maintain proper hygiene of the wound till complete healing. Patients were evaluated weekly for 1 month to assess the wound healing. Injection triamcinolone acetonide 40 mg/ml was infiltrated around the margins just after surgery and again after a gap of every 3-4 weeks for 6 months. For the first two visits, dose of intralesional triamcinolone acetonide 40 mg/ml was 2 U (0.050 ml) of insulin syringe was injected per cm^2^ area and after that the steroid was injected only at the places where itching or pain or reappearance of keloid tissue (surface elevation) was present. Detailed examination was done after 3 and 6 months of the surgery and size of keloid tissue with local and systemic side effects of corticosteroid were noted. After that, patients were followed up to look for recurrence. Patients were categorized as satisfactorily treated, if there was a thin scar (parchment like) or supple texture and no surface elevation, absence of pain/pruritus and absence of tracts.[9] If not, patient was considered as treatment failure.

## RESULTS

A total of 28 patients with 35 keloid lesions were treated with CO_2_ laser followed by intralesional triamcinolone acetonide injection infiltrated at the margins of treated keloid. Nineteen (68%) were males and nine (32%) females. Twenty-eight keloids were on chest, one was on ankle, three were on right flank, one was on abdomen, one was on left arm and one was on lateral side of right thigh. Seventeen keloids (51.42%) were asymptomatic; however, 17 (45.71%) had itching and pain and one (2.85%) had only pain as symptoms. After 21 days of CO_2_ laser ablation, healing with erythematous scar tissue was seen in 33 keloids (94%); however, two keloids became secondarily infected and hence healing was delayed. There was no or minimal pain after 7 days of procedure. Thirteen patients with 17 keloids came for follow-up regularly and completed the protocol and took upto eight intralesional triamcinolone acetonide 40 mg/ml 3-4 weeks apart. Only two patients showed recurrence, one with marginal elevation and another with 70% recurrence. Rest 11 patients having 15 keloids showed complete flattening without any recurrence after a follow-up of 1 year [Figures 3a and b, 4a and b]. Eight keloids showed no local side effects of intralesional steroid, three developed telangiectasias, five developed depigmentation and one developed atrophy. Intralesional steroid dose, which was 10 U (1 ml=40 U of insulin syringe, 1 U=0.025 ml) or 0.25 ml of 40 mg/ml of injection triamcinolone acetonide on an average at first two follow-ups, decreased to an average of less than 2 U or 0.050 ml of injection triamcinolone acetonide at the end of 6 months period [Figure 5]. Ten patients with 12 keloids did not come for follow-up regularly for intralesional steroid and hence did not complete the protocol. In this group, three keloids did not show any recurrence, five keloids showed 100% recurrence and remaining four keloids showed 10-70% recurrence after 1 year of follow-up [Table 1]. An average of three doses of intralesional steroids was taken by these irregular patients in comparison with eight doses of intralesional steroids of regular patients. Five patients were lost to follow-up.

**Table 1:** Follow-up log of patients at 6 months and 1 year after procedure

Id no	age (years)	Sex	Size (cm^3^)	after 6 months of procedure			after 1 year of procedure			Remark
				Recurrence	Local S/E	Systemic S/E	Recurrence	Local S/E	Systemic S/E	
1	22	M	2×1×0.5	1	T	No	1	T	No	Regular F/U
2	20	M	4×2×0.5	1	No	No	6	No	No	Irregular F/U
3	20	M	2.5×2×0.5	1	No	No	6	No	No	Irregular F/U
4	20	M	2×2×0.5	1	No	No	6	No	No	Irregular F/U
5	35	F	5×3×1.0	0	No	No	0	No	No	Regular F/U
6	32	M	4×3×0.5	0	T	No	0	T	No	Regular F/U
7	32	M	2×0.5×0.5	0	No	No	0	No	No	Regular F/U
8	50	M	10×3×0.5	0	No	No	0	No	No	Regular F/U
9	16	F	4×2×0.5	0	A/T	No	0	A/T	No	Regular F/U
10	60	F	8×3×0.5	0	No	No	0	No	No	Regular F/U
11	40	M	6×4×1.0	3	No	No	4	No	No	Regular F/U
12	25	F	3×2×0.5	0	D	No	0	D	No	Regular F/U
13	42	M	6×4×0.5	0	T	No	0	T	No	Regular F/U
14	60	M	10×2×1.5	6	No	No	6	No	No	Irregular F/U
15	28	F	8×4×0.5	0	No	No	0	No	No	Regular F/U
16	42	M	5×3×1.5	2	No	No	2	No	No	Irregular F/U
17	38	M	4×3×1.5	3	No	No	4	No	No	Irregular F/U
18	40	M	4×4×1.5	3	No	No	3	No	No	Irregular F/U
19	30	M	5×4×0.5	1	No	No	1	No	No	Irregular F/U
20	28	M	3×3×0.5	0	No	No	0	No	No	Irregular F/U
23	35	M	3×2×1.5	0	No	No	0	No	No	Irregular F/U
26	26	F	4×1×0.5	0	A	No	0	A	No	Regular F/U
27	26	F	4×1×0.5	0	A	No	0	A	No	Regular F/U
28	30	F	3×0.5×0.5	0	No	No	0	No	No	Irregular F/U
29	25	F	3×1×0.5	0	A	No	0	A	No	Regular F/U
30	18	M	8×6×3	3	No	No	6	No	No	Irregular F/U
33	30	M	3×3×0.5	0	A/T	No	0	A/T	No	Regular F/U
34	50	M	6×2×1	0	No	No	0	No	No	Regular F/U
35	50	M	4×2×1	0	No	No	0	No	No	Regular F/U

Recurrence of keloid from initial size 1-10%=1, 11-30%=2, 31-50%=3, 51-70%=4, 71-90%=5, 91-100%=6, A=atrophy, T=telangiectasia, D=depigmentation, regular F/U=patients taken 3-4 weekly intralesional steroids for 6 months, irregular F/U=patients not taken 3-4 weekly intralesional steroids

**Figure 3a:**
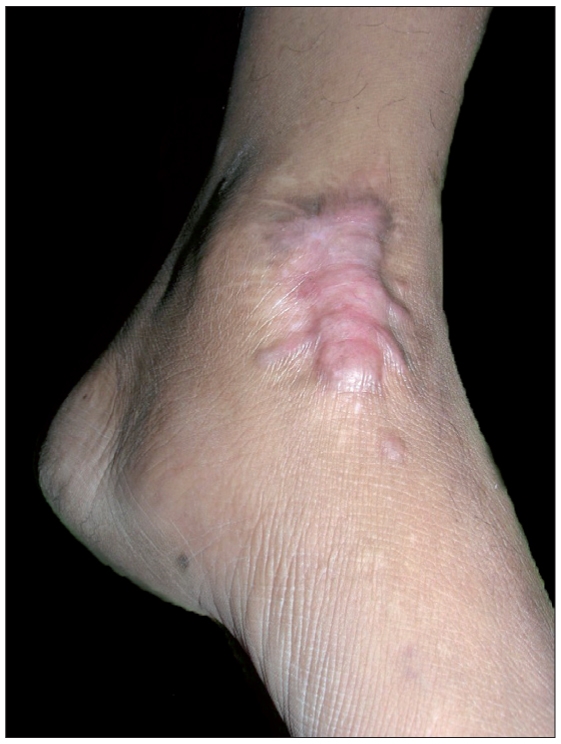
Keloid over ankle prior to treatment

**Figure 3b:**
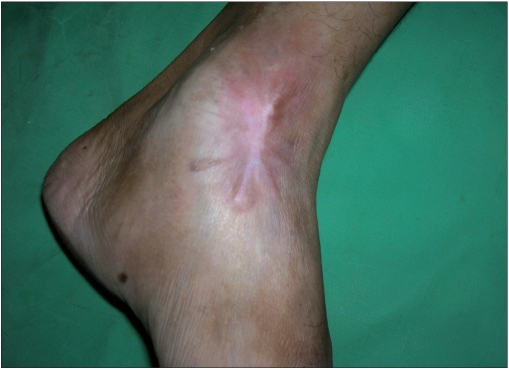
Post-treatment after 1 year of surgery

**Figure 4a:**
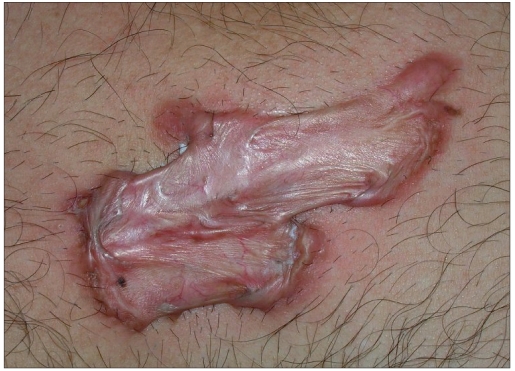
Keloid over chest prior to treatment

**Figure 4b:**
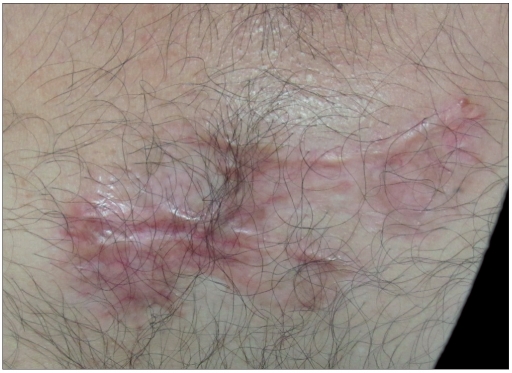
Post-treatment after 1 year of surgery

**Figure 5:**
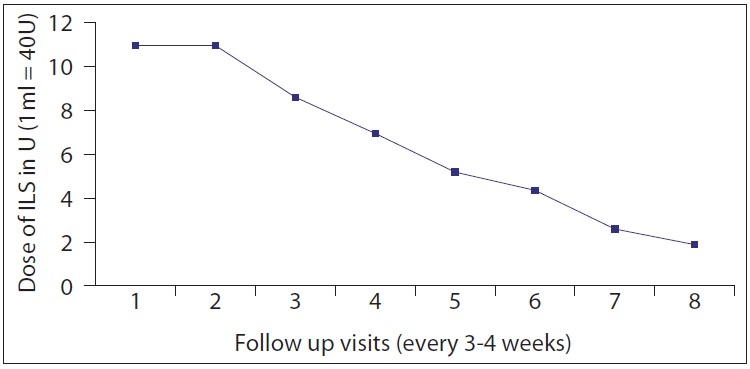
Graph showing average dose of intralesional steroid (ILS) on follow-up visits

We compared the results of regular follow-up patients with the irregular follow-up patients who had not taken intralesional steroid 3-4 weekly as the study does not have a control group.

Data was entered in a 2×2 table. Because the values were less than 5, we applied Fisher’s exact test for *p*-value.

*p*-value was 0.0008976 (<0.005) which was significant (degree of freedom=1).

**Table:** 

	Follow-up at 1 year	
	Regular patients	Irregular patients
Recurrence of	Present	2	9
keloid tissue	Absent	2	9

Symptomatic relief was noted in most patients who took intralesional steroid regularly. Secondary complications were mild-to-moderate, manageable and acceptable to patients. Intralesional steroid injections during follow-up visits were found to be relatively painless probably because they are delivered in periphery or in soft-to-firm reappearing keloid tissue.

## DISCUSSION

Keloids are benign hyperplasias of dermal collagen which may or may not be preceded by injury in susceptible persons. They are refractory to treatment most of the times. Carbon dioxide laser emits far infrared light of wavelength 10600 nm.[10] It uses water as chromophore and vaporizes water in keloid tissue, thus causing damage to tissue.[11] For excision, a point beam of size 0.1 mm diameter with power of 15-25 W is recommended.[12] For vaporization, a point beam of size 1-3 mm with low-power settings (10 W) is recommended.[12] Our machine had maximum power output of 15 W. Superpulse delivers a chain of rapid, short, 200-400 microsecond pulses with high-peak power, which reduces collateral thermal damage.[13] Apfelberg in 1984 published results of CO_2_ and argon laser ablation of keloid. In his study, he treated 13 patients with keloid, ablating them with CO _2_ laser.[6] Except one, all cases recurred after a follow-up of minimum 6 months. He ablated keloid tissue with Argon laser creating multiple punctures throughout the depth of tissue.[6] We used this technique with CO _2_ laser and found that it is an easier approach for very large, hard keloids and gives very good hemostasis. Two or more sittings may be required to ablate the whole keloid tissue with this multiple puncture technique. Again in 1989, the same author published another study, in which he treated seven patients with nine keloids excising them with CO_2_ laser. However, eight of the nine keloids recurred after a follow-up of 6 to 22 months.[9] In none of his studies, he used any other therapy after healing of the lesions to stop the progress of keloid. In 1991, Norris J.E., published his results of CO_2_ laser ablation of keloid. In this retrospective study, he focused on 31 patients with one or more keloids, 23 of whom were available for follow-up after CO_2_ laser excision. One patient had no recurrence of her keloid after CO_2_ laser excision, nine patients required steroids to suppress recurrences, and 13 patients were considered failures.[14] Because of the problem of recurrences in keloid, Stucker and Shaw in 1992 delivered 40 mg/ml of injection triamcinolone acetonide, 150 mg of hyaluronidase and 2% lignocaine intralesionally using a dermajet injector 3-4 weeks apart to stop early recurrences developing after resection with CO _2_ laser. Patients were followed up for the next 2 years and a control rate of 84% was achieved by them.[15] In 2002, Verma published his experiences with CO_2_ laser.[16] He treated 14 patients out of which 12 patients were available for follow-up. CO_2_ laser with a power setting of 20 W was used in continuous mode to vaporize/excise the lesions. In all, except one patient, the healing started by 4 weeks and complete healing of the treatment area occurred within 8 weeks. Four patients with poor response to CO_2_ laser were given 40 mg/ml intralesional triamcinolone acetonide which resulted in a better response in these patients. One patient had depigmentation of the treated area. There were no side effects in other patients.[16]

We used the same approach and found that the patients who were regular for follow-up and took intralesional steroids at every 3-4 weeks for 6 months had complete flattening of keloid tissue. There was no reappearance of keloid tissue after a follow-up of 6 months of therapy in 11 patients with 15 keloids who were regular and completed the protocol. There was no or minimum pain after 7 days of procedure. Wound healed with erythematous scar tissue within 3 weeks of procedure. Since we did not have a control group, we only compared the results of patients who followed up regularly and took intralesional steroid injections every 3-4 weeks with those who did not. We found that the difference in recurrence rate of regular and irregular patients was statistically significant.

This indicates that only CO_2_ laser ablation is not sufficient for halting the pathogenesis of keloid formation. It requires regular 3-4 weekly intralesional steroid for a period of 6 months after ablation with CO_2_ laser.

## CONCLUSION

Ablation with CO_2_ laser followed by regular 3-4 weekly intralesional steroid for 6 months, is a satisfactory approach for keloid management. Recurrence rate in the first 6 months is low. Further studies with larger number of patients and longer follow-up are required to further assess efficacy of CO_2_ laser with intralesional steroid in treatment of keloids.
